# The Oldest Gibbon Fossil (Hylobatidae) from Insular Southeast Asia: Evidence from Trinil, (East Java, Indonesia), Lower/Middle Pleistocene

**DOI:** 10.1371/journal.pone.0099531

**Published:** 2014-06-10

**Authors:** Thomas Ingicco, John de Vos, O. Frank Huffman

**Affiliations:** 1 Archaeological Studies Program, University of the Philippines, Quezon City, Diliman, Philippines; 2 Département de Préhistoire and UMR 7194, MNHN/CNRS, Paris, France; 3 Naturalis Biodiversity Center, Leiden, The Netherlands; 4 Department of Anthropology, University of Texas at Austin, Austin, Texas, United States of America; University of Florence, Italy

## Abstract

A fossil femur excavated by Eugène Dubois between 1891–1900 in the Lower/Middle Pleistocene bonebed of the Trinil site (Java, Indonesia) was recognised by us as that of a Hylobatidae. The specimen, Trinil 5703 of the Dubois Collection (Leiden, The Netherlands), has the same distinctive form of fossilization that is seen in many of the bonebed fossils from Trinil in the collection. Anatomical comparison of Trinil 5703 to a sample of carnivore and primate femora, supported by morphometric analyses, lead to the attribution of the fossil to gibbon. Trinil 5703 therefore provides the oldest insular record of this clade, one of the oldest known Hylobatidae fossils from Southeast Asia. Because living Hylobatidae only inhabit evergreen rain forests, the paleoenvironment within the river drainage in the greater Trinil area evidently included forests of this kind during the Lower/Middle Pleistocene as revealed here.

## Introduction

The site of Trinil (East Java, Indonesia) is famous for being the locality that yielded the type specimen of *Pithecanthropus erectus* Dubois ([1], mostly cited as 1894), known as *Homo erectus* since Mayr [2]. In this work, we report the discovery of a gibbon femur in the Trinil fossils assemblage, preserved in the Dubois Collection at the NCB Naturalis, Leiden (the Netherlands). The fossil was catalogued in the 1930s under the supervision of Dubois (Shipman 2001). The fossil was packed in a separate box labeled: “*Semnopithecus* - (Kleine soort) – l. femur” [Translated: *Semnopithecus* - (small species) – left femur]. Following examination of the specimen it became clear that it was attributable to a Hylobatidae, rather than to *Semnopithecus*.

Small apes are very scarce in the fossil record [3], making the reporting of even fragmentary new discoveries valuable. Moreover, as we show here, Trinil 5703 represents the oldest known presence of small apes in insular Southeast Asia [SEA], and provides further evidence of ever-wet forest habitat in an area of *Homo erectus* occupation. After describing the fossil femur anatomically, we compare it with SEA mammals of similar size in order to establish the taxonomic attribution of Trinil 5703.

Since its first description, two major amendments have been made to the Trinil fauna. First, Badoux [4] separated the fossils collected by von Koenigswald in the Punung area from the ones collected in Trinil, resulting in the creation of the distinct Punung fauna, considered to be Upper-Middle Pleistocene. Second, on the basis of excavation archives, de Vos and Sondaar [5] and de Vos et al. [6] reviewed the stratigraphic provenance of the fossils excavated at Trinil by Dubois in 1891–1900 and the Selenka expedition in 1906–1908, concluding that the bulk of the specimens came from a single bonebed named the *Hauptknochenschicht*. Following de Vos and Sondaar [5], the bonebed is referred to as Trinil H.K. in this paper. De Vos et al. [6] defined a biostratigraphic unit, the Trinil H.K. fauna, from the assemblage of vertebrate taxa, including *Homo erectus*, originating from this bonebed.

### 1.1. Biostratigraphy

De Vos et al. [6] also reinterpreted the biostratigraphic position of the so-called Jetis fauna, which von Koenigswald [7] considered to be older than the Trinil fauna, but which according to de Vos et al. [6] is younger than their Trinil H.K. fauna. The Jetis fauna was further subsumed into a Kedung Brubus fauna, proposed and defined in the same paper. De Vos et al. [6] further defined Satir, Ci Saat, Ngandong, Punung and Wajak faunal association which have been highly influenced by eustatic fluctuations resulting in cyclic connections of Java to mainland Southeast Asia allowing migrations compared to endemic evolutions during insular conditions [8].

These new biostratigraphic zones (biozones) have been dated radiometrically [9]–[12] as follows: the Satir fauna at 1.5 myr, Ci Saat fauna at 1.2 myr, Trinil H.K. fauna at 1 myr, Kedung Brubus fauna at 0.8 myr, Ngandong fauna at 0.2–0.07 myr, Punung fauna at 0.12 myr, and Wajak fauna as Late Pleistocene to Holocene. The dates continue to be debated [13], [14], and especially so, the age of the Ngandong fauna [15]–[20]. No radiometric dating of Trinil bonebed has been reported, the age of 1 Myr for Trinil H.K. was mostly established on the basis of biostratigraphic correlation with assemblages collected at the Sangiran Dome, Java [*e.g.* 10]. Here, we use the coupled ESR/U-series and ^40^Ar/^39^Ar date of 0.8 myr that is obtained from the fossil-bearing beds at Ngebung 2, recently excavated by modern archaeological methods and referred to as Final Trinil H.K. fauna [21]–[23]. The biostratigraphical correlation of the Ngebung 2 fauna with Trinil H.K. is based on (a) the presence of *Stegodon trigonocephalus* and absence of *Elephas hysudrindicus* [24] which makes it older than the Kedung Brubus biozone where this latter species first appears [6], [25] and (b) the presence of the cervid *Axis lydekkeri ngebungensis* that differs on subspecies level with *Axis lydekkeri* and has therefore been used to define this association as a Final Trinil H.K. fauna [22]. The radiometric dates obtained at Ngebung 2 are consistent with the previous estimate age of ∼1 myr for the Trinil bonebed by von Koenigswald [26] and Leinders et al. [10]. We therefore take 0.8 myr as the most reliable current age estimate for the Trinil H.K. and thus Trinil 5703.

Apart from the hominin remains, Trinil H.K. yielded the oldest undisputed primates in insular Southeast Asia [27], [28]. These are the robust Colobinae *Trachypithecus auratus robustus* and the Cercopithecinae *Macaca fascicularis* (in Hooijer [29]). Von Koenigswald [30, p.63–64, 209] mentions the presence of the gibbon *Hylobates* and the siamang *Symphalangus*, and illustrates a molar of *Hylobates moloch* (formerly *H. leuciscus*) discovered in the Jetis biozone at Sangiran Dome. Hooijer [31] also mentions the presence of *Hylobates* in the Jetis and Trinil faunas (*sensu* von Koenigswald [7]). Later on, while describing the Punung fauna, Badoux [4] reallocated all the Hylobatidae formerly associated to the Trinil fauna (40 teeth) to this new biozone. Therefore, the oldest published Hylobatidae are from the Punung fauna of Java (128 Ka; [11], [32]). The two fossils collected by von Koenigswald [30] in Sangiran and assigned by him to the Jetis fauna, disappeared in the biostratigraphy of de Vos and Sondaar [5]. Nevertheless, because they are reported coming from the Sangiran Dome, those two Hylobatidae should be older than the Punung fauna.

### 1.2. Stratigraphy of the trinil site

**Figure 1:**
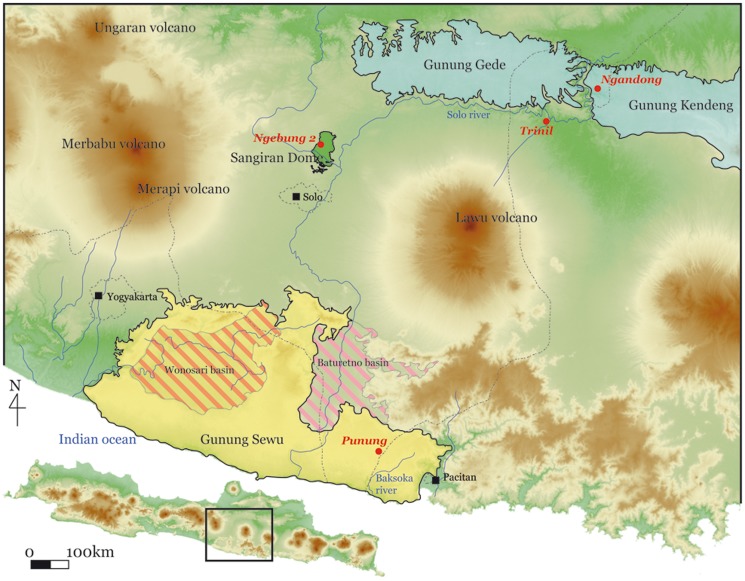
Map of Central Java pointing to the site of Trinil and other archaeological and paleontological sites mentioned in the text. This map was built up with GRASS open-source software from open access SRTM data [86] available from http://srtm.csi.cgiar.org.

The Trinil H.K. bonebed lies near the base of the Kabuh Formation where it is in contact with the Pucangan Formation [33]. The two formations dip gently southward in the vicinity of Trinil, as bedrock strata generally do along the southern edge of the Kendeng Hills (Figure1). The bonebed is exposed along the Solo River in the vicinity of Trinil due to river entrenchment of ∼10 m into the bedrock formations [34]–[36]. The Pucangan Formation exposed near the site is primarily tuffaceous conglomerate (sedimentary “breccia”) deposited by volcanic mudflows (lahars). The overlying Trinil H.K. is a volcaniclastic lens, deposited fluvially and filling a paleotopographic swale at the top of the Pucangan Formation; while the lens extends laterally for several hundred meters, it is only about a meter or two thick [34], [36]–[38].

Lithologically, the Trinil H.K. is indurated gravelly sandstone. The stratum is very poorly sorted granulometrically and composed largely of fresh volcaniclastic materials. Besides carrying tens-of-thousands of well-preserved vertebrate fossils, including the remains of both terrestrial and aquatic animals, such as crocodile and fish [35], [5], [39], the Trinil H.K. also contained plant fossils, including broken tree trunks, and well-preserved fresh-water molluscs [40], [37], [41], [42].

Given the lithological and paleontological features of the Trinil H.K., it most likely accumulated from muddy flood waters that originated as a lahar on an active stratovolcano tens of kilometers to the south, descended the drainage to join a large river, passed through several biotopes, including tropical forest and grass-dominated areas, and inundated a swampy lowland [43], [37], [42], [44]. The lowland was bordered a few kilometres north of Trinil by the marl bedrock of the Kendeng Hills, which contributed little clastic sediment to the bonebed relative to the more distant volcanic source terrain on the south [45] (see the paleogeographic map of Huffman and Zaim [46]).

**Figure 2:**
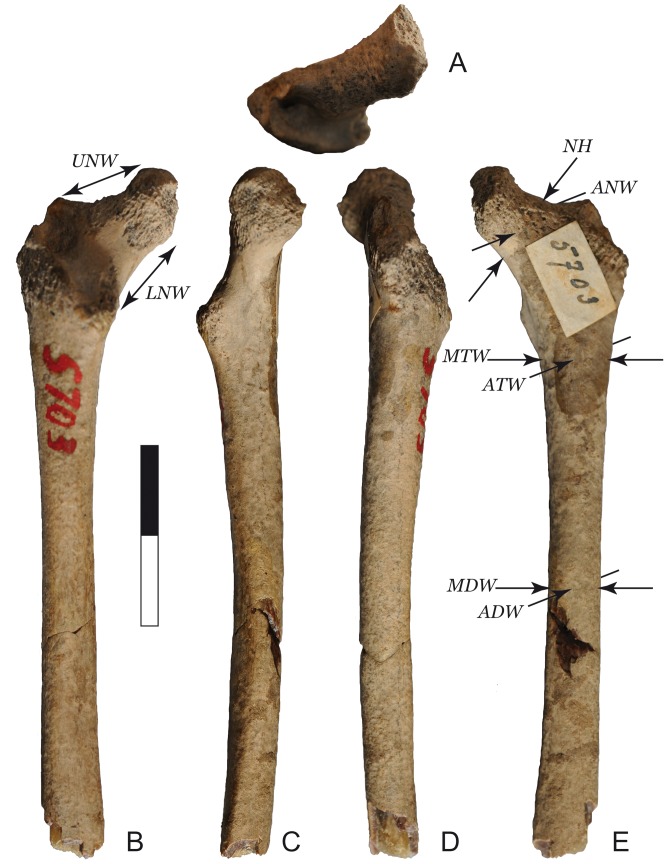
View of Trinil 5703 femur. **A**: Proximal view; **B**: Posterior view; **C**: Medial view; **D**: Lateral view; **E**: Anterior view.

**Figure 3:**
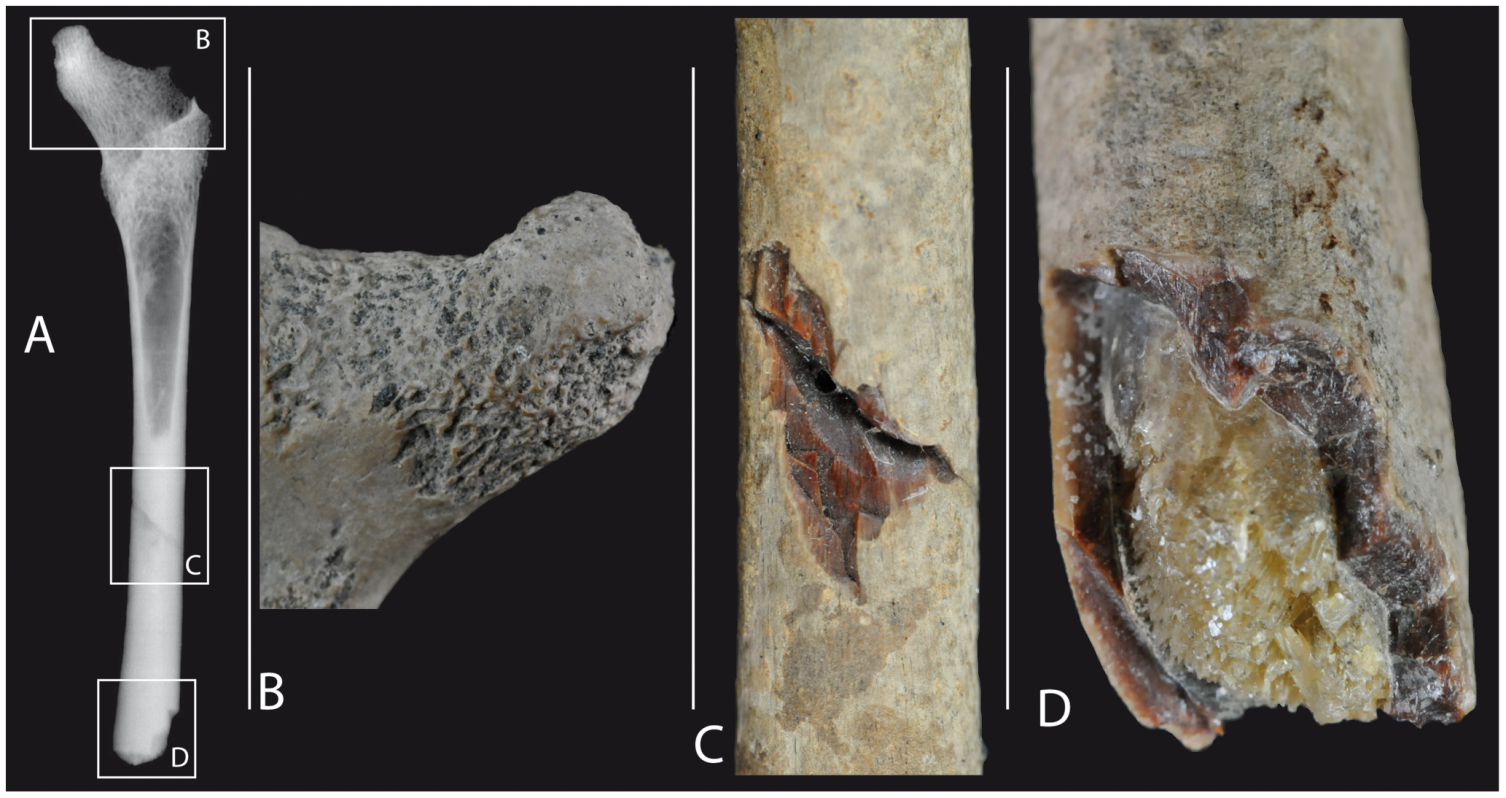
Taphonomic details of Trinil 5703. A: radiograph in anterior view showing an opaque filling in the distal portion of the preserved medullar cavity; **B**: detail of the neck in posterior view, showing fluvial abrasion in weakly acid conditions; **C**: detail of the refitted break of the shaft that we attribute to breakage during excavation; **D**: detail of the distal break of the shaft, which is likely also excavation damage, and the calcitic filling inside the medullar cavity, as also seen on the radiograph.

Specimen 5703 is attributable to the Trinil H.K. bonebed, and thus this paleoenvironmental setting, for several reasons. First, Trinil 5703 has the same distinctive form of fossilization that is seen in many of the bonebed fossils of the Collection. The fossilization gives fossils like Trinil 5703 a brown, near glassy, appearance on surfaces where the cortical bone is freshly broken (Figure 2, 3c and 3d). Second, most of the Trinil fossils in the Dubois Collection originate from the Trinil H.K. The yellowish colour of the calcite crystals that have grown inside the femur medullar cavity further agrees with an origin of the fossil from the Trinil H.K. bonebed (Figure 3d).

### 1.3. Preservation

Trinil 5703 is a partial left femur that preserves an incomplete proximal epiphysis and a portion of the shaft that is broken just below the nutrient foramen. Assuming that the specimen is from a fully-grown individual, as we establish below, Trinil 5703 therefore preserves 60% of its biomechanical length [47] (Figure2). The extremity of the lesser trochanter is missing, and the head and the greater trochanter are mostly lacking. Only a small portion of the head on the posterior aspect and part of the lateral aspect of the greater trochanter are preserved. The line of fusion of the head is preserved, however, and the fusion of the epiphysis and diaphysis are complete.

Considering that the proximal epiphysis fuses later than the distal epiphysis in apes, the length growth of the femur had ended. The developmental age is nevertheless uncertain, because epiphysation of the hip occurs early in the life of gibbons and other primates [48], [49], much before the eruption of the third molar, which is considered to be the boundary marker between youth and adulthood. Whether an adult or sub-adult by this criterium, the individual would have experienced no further growth along the epiphysed proximal end of its femur and had reached a practically full-grown stature.

Trinil 5703 exhibits damage attributable to both pre-burial events and excavation loss. Two fresh breaks occur on the shaft (Figure 3C and D). One broke the fossil in two. They were glued together decades ago and appear to refit perfectly. The edges along the break are sharp and not abraded, leading us to conclude that the fracture occurred during or after excavation. A chip of the shaft has been lost from along this break, and the gap reveals a brown, near glassy appearance of the fossilized cortical bone that from our own observations is typical of Trinil H.K. specimens in the Dubois Collection. The second fresh break occurs on the distal extremity of the femoral shaft, and is weakly abraded (Figure 3D), indicating the fracture was not strongly damaged during the fluvial transport it underwent. The distal break of the shaft reveals filling of the medullar cavity by well-crystalized, transluscent mineral that appears to be calcite (Figure 3D). The radiograph of the fossil shows that the calcite fills half of the preserved portion of the shaft (Figure 3A).

Other pre-burial taphonomic processes can be reconstructed from study of the surface of Trinil 5703. The cortical surface of the neck is damaged to the point that the trabeculae are prominently visible, except on the distal face. The femoral head and the greater and lesser trochanters are damaged to a similar degree (Figure 3B). The loss of bone surface of Trinil 5703 might well be due to wet and weakly acid conditions while the femur was at the ground surface after skeletonization but before transport because weathering rates are very fast in the tropics [50]–[51].

## Materials and Methods

The specimen we studied and describe in this paper is a a proximal femur labeled 5703 and curated in the Dubois Collection of the National Biodiversity Center Naturalis in Leiden, The Netherlands. Even though the fragmentary nature of Trinil 5703 precludes the acquisition of many classical anatomical measurements, some detailed morphological comparisons are possible, and although the fossil cannot be identified to a species level, its attribution to Hylobatidae is evident.

**Figure 4:**
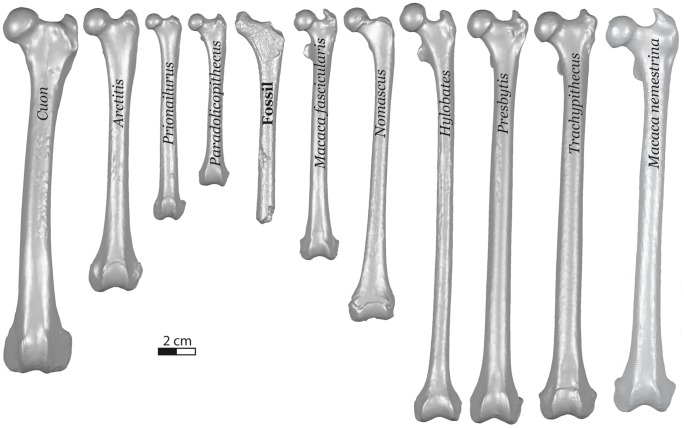
Comparison of the Trinil 5703 fossil femur (center) in anterior view with the femora of Southeast Asian carnivores (on the left side of Trinil 5703) and primates (on the right side of Trinil 5703). 3D models of the specimens obtained by surface scanning with a Nextengine Desktop 3D scanner.

We first visually compared Trinil 5703 with femora of Southeast Asian carnivore and primate species of body sizes similar to gibbon, thus excluding very large taxa (*Panthera* and *Pongo*) and very small ones (*Tarsius* and *Nycticebus*) (Figure 4). The Trinil fauna contains three carnivores, *Panthera tigris*, *Prionailurus bengalensis* and *Mececyon trinilensis*, and two primates, *Trachypithecus auratus robustus* and *Macaca fascicularis*. Modern examples of *Prionailurus bengalensis, Trachypithecus auratus robustus*, and *Macaca fascicularis* were included in our sample. *Mececyon* is an extinct canid closely related to the extant *Lycaon* which is of similar shape with *Cuon alpinus* which as available to us. We therefore used the living species in our analyses.

We further compared our measurements on Trinil 5703 with those taken from the femora of extant Southeast Asian primates housed at the Muséum national d'Histoire naturelle (Paris, France) and the Naturalis Biodiversity Center (Leiden, The Netherlands), again excluding the very small and very large genera. This sample comprises 70 adults, 10 sub-adults and 6 juveniles when available in the collections for a total of 86 individuals from nine genera and 18 species (Table 1). For some of our analyses, we grouped the species into four natural clades, namely: small apes (*Bunopithecus*, *Hylobates*, *Nomascus*, *Symphalangus*), langurs (*Presbytis*, *Trachypithecus*) odd-nosed monkeys (*Nasalis*, *Rhinopithecus*) and macaques (*Macaca fascicularis* and *Macaca nemestrina*).

**Table 1:** List of species used in this study.

Group	Genera	Species	N
Lesser apes	*Bunopithecus*	*B. hoolock*	2
	*Hylobates*	*H. lar*	6
		*H. moloch*	7
		*H. muelleri*	10
	*Nomascus*	*N. concolor*	3
		*N. gabriellae*	3
		*N. leucogenys*	17
	*Symphalangus*	*S. syndactilus*	1
Langurs	*Presbytis*	*P. chrysomelas*	4
		*P. hosei*	2
		*P. rubicunda*	2
	*Trachypithecus*	*T. auratus*	4
		*T. cristatus*	2
		*T. johnii*	2
		*T. phayrei*	2
Odd-nosed	*Nasalis*	*N. larvatus*	5
	*Pygathrix*	*P. naemeus*	1
Macaques	*Macaca*	*M. fascicularis*	17
		*M. nemestrina*	11

Eight linear measurements, taken with a digital calliper (Mitutoyo Digimatic Calliper with an accuracy of 0.01 mm), are used for metrical comparisons. The length of the neck was measured from the line of fusion of the head to the lesser trochanter (referred to as the LNW distance) and from the line of fusion of the head to the medial aspect of the greater trochanter (UNW). The other six measurements were the antero-posterior width of the neck (ANW), the minimum proximo-distal height of the neck (NH), the antero-posterior and medio-lateral shaft widths just below the lesser trochanter (ATW and MTW, respectively), and the maximum antero-posterior and corresponding medio-lateral shaft widths (ADW and MDW, respectively). We also measured the total length of the femur in our comparative sample in order to estimate the total length (TL) of Trinil 5703 by means of linear regression equations.

Our measurements of the specimens and the fossils (Table 2) are presented as bivariate plots. We estimate the original length of Trinil 5703 femur from our own calculated allometric regressions. This is because first, only [52] provided regressions for the femur of either small apes or monkeys, and second, when applied to our comparative material, none of those equations allowed us to retrieve or approximate the true femur lengths, so we developed our own equations. The proximo-distal femoral neck height (NH), because it is preserved in Trinil 5703, has been used to calculate allometric regressions of the femur length once log-transformed, as proposed by [52]. Equations are presented for each three of the four natural clades established earlier, small apes, langurs and macaques (Table 3). Because Trinil 5703 is clearly out of the range both morphological and morphometrical of odd-nosed monkeys, we did not calculate any allometric regression. Femur length for Trinil 5703 was then estimated using each of the three equations from the logarithm of the neck length. We therefore obtained three different femur lengths for the fossil that we compared one by one with the respective femur length range of each primate clade.

**Table 2:** Comparative measurements of Trinil 5703 femur with primate samples (all measurements are in mm).

	LNW	UNW	ANW	NH	ATW	MTW	ADW	MDW	TL
Trinil	9.93	10.44	7.53	9.86	11.15	9.97	7.53	7.18	-
**Small apes (n = 49)**									
Median	10.51	6.31	8.82	10.88	12.62	10.26	10	10.3	193
Mean	10.60	6.27	8.96	10.93	12.71	10.26	9.72	10.06	190.9
Std dev	2.072	1.350	1.332	1.361	1.532	1.394	1.369	1.441	20.096
Min.	6.68	3.95	6.5	8.57	10.11	7.95	7.11	6.93	145.7
Max.	15.02	9.79	11.66	13.35	15.19	13.15	12.02	12.57	221.1
**Langurs (n = 18)**									
Median	8.68	5.43	9.17	13.49	12.55	11.16	10.11	10.39	193.7
Mean	8.89	5.04	9.47	13.31	11.91	11.29	9.84	10.32	196.8
Std dev	2.47	1.596	1.834	1.519	2.1	1.9	1.72	1.662	19.478
Min.	4.46	1.86	7.12	11.01	7.78	7.7	6.35	6.99	175.6
Max.	13.96	8	14.32	15.64	14.52	13.77	11.89	12.78	220.8
**Odd-nosed (n = 6)**									
Median	12.38	6.94	13.6	18.27	14.69	14.46	12.97	13.31	233.9
Mean	12.67	7.335	14.25	18.26	15.16	15.38	13.43	13.87	233.6
Std dev	1.56	1.230	2.822	2.186	1.578	1.595	1.246	1.316	18.136
Min.	11.18	6	10.68	15.97	13.1	14.15	12.35	12.52	217
Max.	14.44	8.95	17.66	20.54	17.33	17.65	15.14	15.91	249.6
**Macaques (n = 28)**									
Median	10.19	7.33	10.16	11.42	11.59	10.85	10.43	10.71	158.2
Mean	10.05	7.06	9.91	11.54	11.69	11.12	10.4	10.84	158
Std dev	1.928	1.273	1.546	1.847	2.029	1.685	2.08	1.934	29.637
Min.	6.77	3.95	7.5	8.67	8.53	8.09	7.29	7.3	117.4
Max.	13.37	11.66	12.98	15.17	15.85	14.05	14.28	13.95	209.3

**Table 3:** Allometric regression equations for the different natural clades studied: small apes, langurs and macaques, used to estimate the original length of the femur based on the log-transformed Neck height.

Equation	y	x	N	correlation coeficient	Standard error of the estimate	Slope	95% confidence interval	Intercept	95% confidence interval	Trinil
**Small apes**	ln neck height	ln total length	49	0.796	0.091	0.709	0.524–0.894	3.554	3.112–3.996	177
**Macaques**	ln neck height	ln total length	28	0.94	0.234	1.110	0.910–1.31	2.344	1.857–2.831	132
**Langurs**	ln neck height	ln total length	18	0.64	0.095	0.549	0.011–1.088	3.859	2.467–5.251	171

The table also indicates the original length of Trinil 5703 estimated by each model.

Because the Javanese Pleistocene is characterized by numerous changes in mammal body size [53]–[55], we applied the log-shape ratios method to our measurements, following methods described by [56], in order to remove size and describe shape only. We computed the log-shape ratios together, first in a principal component analysis (PCA), and second in a linear discriminant analysis (LDA) with cross validation on the four natural clades. The discriminant functions were calculated on the comparative material only, and the discriminant residuals of the fossil were calculated by post-multiplying the log-shape ratios of the fossil by the scores of the discriminant functions then obtained. By making Trinil 5703 a “hold-out” individual we obtained a post-classification for the fossil within one of the four pre-defined natural clades and percentages of Type I error of post-classification per clade (Table 4).

**Table 4:** Confusion matrix of the LDA showing the posterior misclassifications for each natural clade.

	Macaques	Small Apes	Langurs	Odd-nosed
**Macaques**	71	14	11	4
**Small Apes**	8	88	4	0
**Langurs**	28	0	72	0
**Odd-nosed**	50	0	17	33

This table gives an estimate of the risk to commit a type I error when attributing Trinil 5703 to the small Apes, which is the result of the LDA.

Hylobatidae are characterized by their very specialized locomotor behaviour, known as suspensory brachiation. Their anatomy reflects this specialization, especially in particularly slender and elongated limbs. Therefore, two characteristics may be regarded as important in the identification of femurs of small apes, which are the length of the femoral neck and the rectilinear shaft of the femur. Geometric morphometric methods have been successfully applied to long-bone curvature in primates by [57] so we applied it here. Because of the presence of the linea aspera on the posterior aspect of the femur that may complicate the record of the curvature, we decided to focus on the curvature of the anterior aspect. The curvature of the shaft was recorded directly perpendicular to the transverse plan of the femur in three dimensions from the freeware Landmarks (Institute for Data Analysis and Visualization, 2002) on 3D surface scans of the femurs acquired with a Nextengine surface scanner. We obtained a set of ten evenly-space horizontal landmarks on the mid-sagittal plane over the femoral shaft with the first and second points adjacent to the line just below the lesser trochanter and half of the shaft length respectively. This curvature was compared to our sample of primates. We first converted the three dimensional coordinate dataset into two dimensions through PCA on each individual one by one. The PCA scores for the two first dimensions of each individual have then been superimposed through baseline registration method as proposed by [58]. A baseline was defined by the first and last landmarks of each curvature reset to the same fixed coordinate position, while the other landmarks followed this sum of rotation, translation and scaling. Newly obtained ‘x’ coordinates correspond to comparative landmarks along the femoral shaft, and the ‘y’ coordinates summarize the shape information. In this way, the ‘y’ coordinates are treated as variables at comparable positions (row number in the dataset) along the femoral shaft. Thus visual comparison of curvatures from one species to another one is straightforward. We also quantified the distances between Trinil femur shaft curvature and other species by calculating the mean distance between the Bookstein coordinates in baseline unit.

All the analyses were performed with R software [59] and formulae available in [60].

## Results

### 3.1 Identification of Trinil 5703 as a primate

Morphological comparison of Trinil 5703 to femora of Southeast Asia carnivores and primates of similar size indicates clearly that the fossil is a primate (Figure 4). Trinil 5703 is not a carnivore because (a) it is longer than *Pardoxurus* and *Prionailurus*; (b) the shaft and neck are narrower than those of *Arctictis* and *Cuon*; (c) the neck is much longer than in all the carnivores in Figure 4; and (d) the lesser trochanter is oriented more posteriorly than any of the carnivores. Trinil 5703 does have a salient gluteal tuberosity that is also seen in *Paradoxurus*, *Prionailurus* and *Arctictis*.

Trinil 5703 is generally similar to the primates shown in Figure 4: (a) the width of the shaft, both ATW over MTW and ADW over MDW, is within the range of the small apes, langurs and macaques (Table 2); (b) the posterior orientation of the lesser trochanter is within the range of the orientations seen in the small ape specimens; and (c) the estimated original length of Trinil 5703 is within the range of small apes and the small macaque *M. fascicularis*.

**Figure 5:**
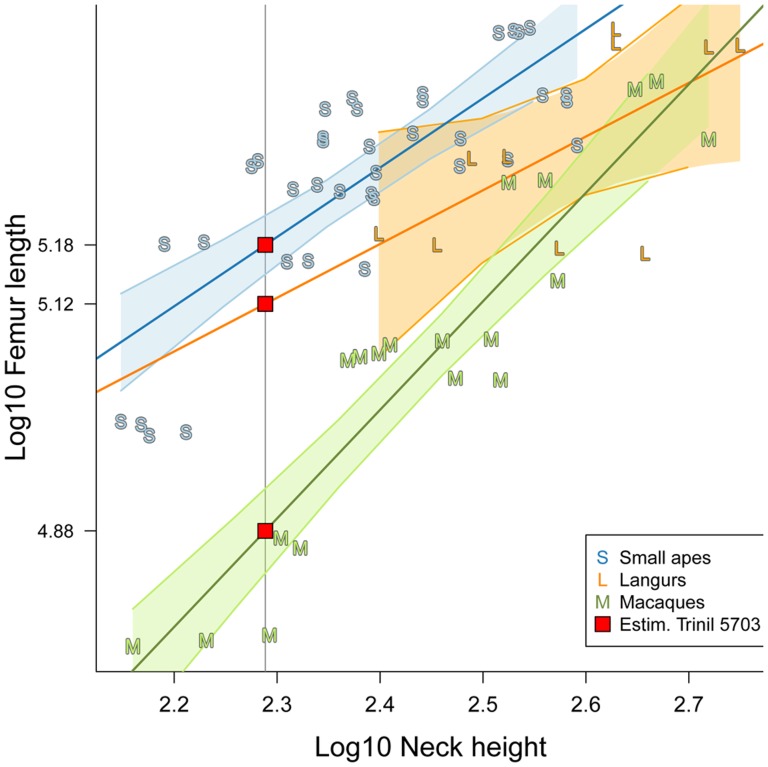
Linear regressions of the log-transformed total length of the femur over the log-transformed femoral neck height for each of the following clades: small apes, langurs and macaques; and estimation by each of the three different models of the original total length of Trinil 5703 (red square) from its log-transformed femoral neck height.

Equations have been calculated from linear regressions of the log-transformed TL over the log-transformed NH for each of the following clades: small apes, langurs and macaques (Figure 5). The equations are given in Table 3. They allowed us to estimate the femur length of Trinil 5703. The regression based on the macaque model produces the lowest estimate (133+/−13 mm), while the largest estimate (178+/−16 mm) is obtained by the small ape model. The regression based on the langur model gives a middle estimate of 171+/−39 mm. This latter estimate is largely out of the range of femur length for the langurs, Trinil 5703 being too small to be either attributed to *Trachypithecus* or *Presbytis* (Figure 5). The femur length estimate obtained by the macaque model clusters Trinil 5703 with the small size *M. fascicularis*. Nevertheless, the preserved portion of Trinil 5703, which corresponds to no more than half of its total length, as we established earlier in the preservation section, is nearly the size of the complete *M. fascicularis* femur (Figure 4). Therefore, the Trinil 5703 femur was unlikely to have had a length comparable to *M. fascicularis*, as predicted by the macaque model. The small ape regression model appears to provide the best estimate for the original length of Trinil 5703 femora (178+/−16 mm).

**Figure 6:**
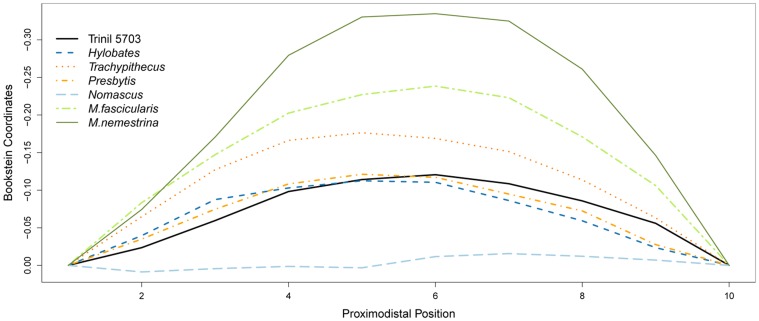
Comparison of Trinil 5703 femoral curvature with other primates through Bookstein baseline regression of ten evenly-space horizontal landmarks on the mid-sagittal plane over the shaft with the first and second points adjacent to the line just below the lesser trochanter and half of the shaft length respectively.

### 3.2 Identification of Trinil 5703 as Hylobatidae

**Figure 7:**
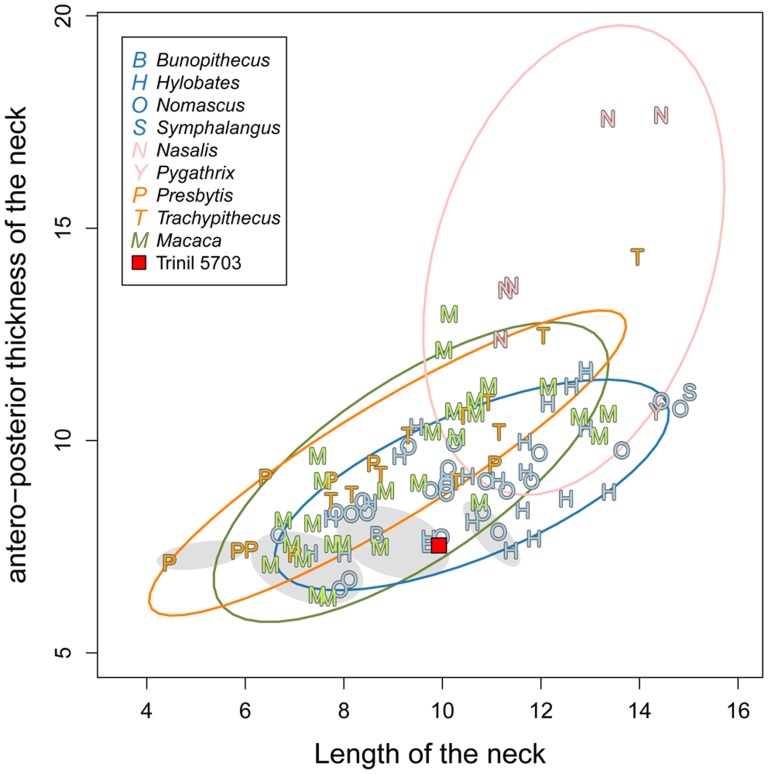
Length and anteroposterior width of the neck of Trinil 5703 and comparative sample (Table 2). Ellipses are 95% confidence interval for each natural clades: small apes, odd-nosed, macaques and langurs. Filled grey ellipses group the juvenile and sub-adult individuals of small apes and odd-nosed monkeys.

**Figure 8:**
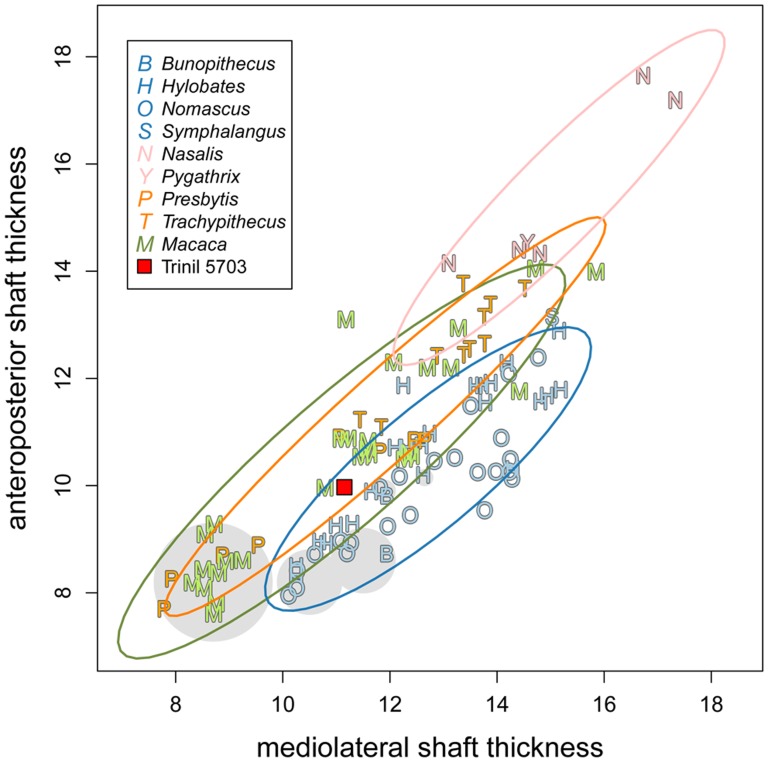
Antero-posterior and mediolateral shaft thickness below the lesser trochanter of Trinil 5703 and comparative sample. Presentation conventions are the same as in Figure 7.

**Figure 9:**
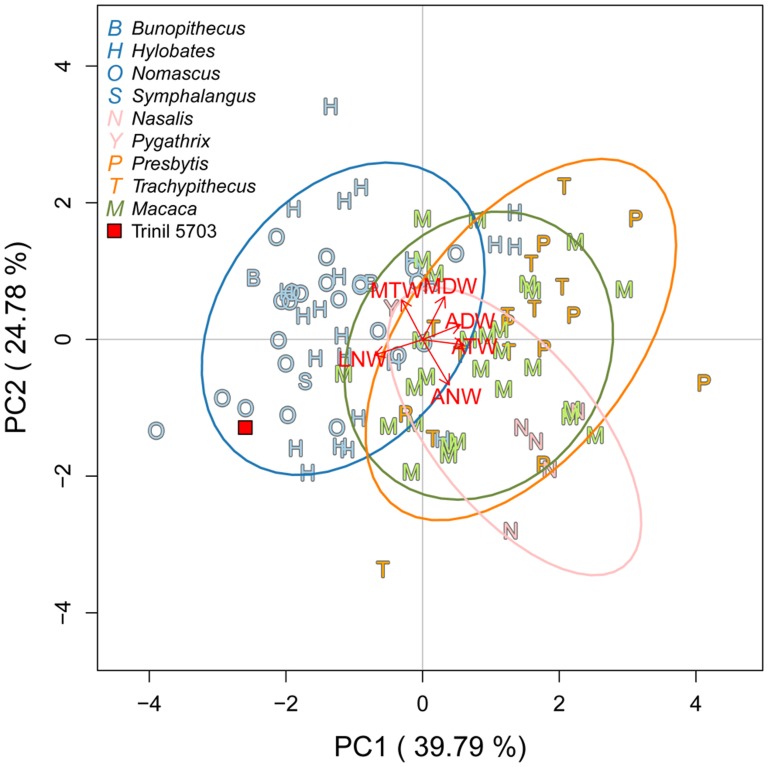
Principal component analysis on log-shape ratios. Presentation conventions are the same as in Figure 7.

**Figure 10:**
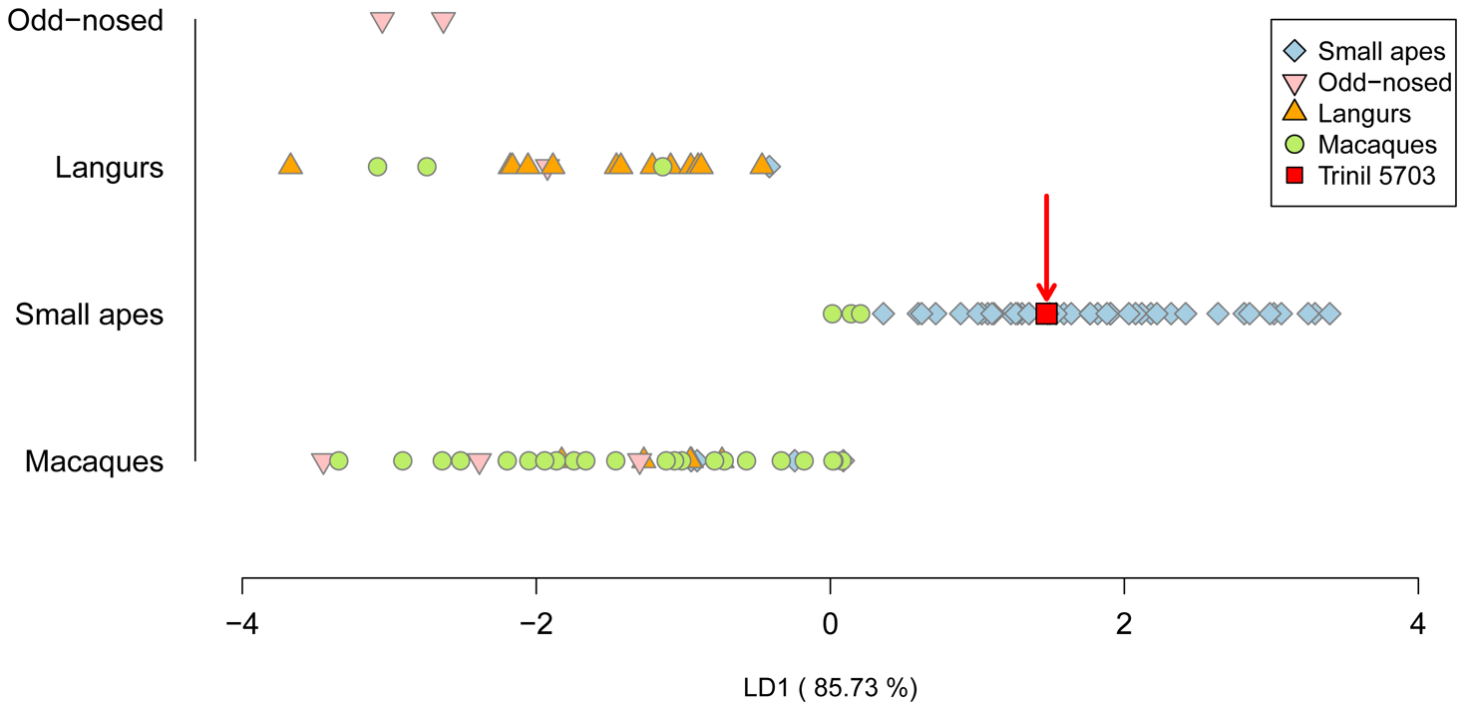
Linear discriminant analysis on log-shape ratios.

Our morphometric analysis of Trinil 5703, summarized in Table 2 and illustrated in Figures 7–10, together with the specimen's overall anatomy (Figure 2–4), indicate that the fossil is a small ape.

Although the femoral head of Trinil 5703 is mostly lacking, the small portion preserved on the posterior aspect likely suggests a globular shape comparable to what is observe in small apes. The head typically extends on the posterior aspect of the neck in Cercopithecidae such as macaques, langurs and odd-nosed monkeys (Figure3B). This characteristic is confirmed in Trinil 5703 with the small portion of the head preserved which clearly overhangs the neck (Figure 2B and 2E). The globular shape allows a high degree of abduction of the hind limb, which is essential in suspensory locomotion.

Trinil 5703 shows an elongated, thin and antero-posteriorly flattened femoral neck, similar to that found in all small apes (Figure7). The proportions of the neck in the fossil are similar to young and sub-adult small apes. Although the neck-shaft angle cannot be measured, the angle formed is obtuse. The anteversion of the neck cannot be estimated here without the distal portion of the femur. There is no tuberculum on the posterior aspect of the neck (Figure2B and 3B). Such a tuberculum is always present in catharrines and occurs in most hominoid taxa, but is usually absent in small apes [61], [52], [62].

The trochanteric fossa of Trinil 5703 femur is small and very rounded (Figure2B). Bacon [60] distinguishes three morphological types of primate trochanteric fossa. A trochanteric fossa located in a slot is characteristic of Strepsirhini, while a large and proximo-distally stretched oval fossa characterizes Cercopithecoidea, such as macaques, langurs and odd-nosed monkeys. A rounded fossa is only recorded in Hominoidea and Atelinae. Bacon [61] interprets the rounded shape of the trochanteric fossa of the kind that we observed in Trinil 5703 as the possibility for a greater mobility of the *ligamentum teres* during brachiating locomotion.

The lesser trochanter is on the posterior face of the shaft of Trinil 5703. The position of the lesser trochanter appears to vary within primate populations [62]. This intraspecific variability also occurs in small apes. A mesial position of the lesser trochanter is reported for Hylobatidae [63], [52]. In counterpart, Piganiol and Olivier [64] indicate that the lesser trochanter is oriented posteriorly in small apes. From our own observations, the position of the lesser trochanter seems to change during the life of the animal from posterior position among young and sub-adults and a mesial position in adults. In Figure2, the lesser trochanter of the adult *Hylobates* is oriented medially but of sub-adult *Nomascus*, posteriorly. Nevertheless, this age-related position of the lesser trochanter has to be confirmed by further studies with larger samples of young individuals, which is beyond the aim of this study. To summarize, the position of the lesser trochanter in Trinil 5703 is within the range of small apes, and may indicate a sub-adult developmental stage.

The shape of Trinil 5703 is typical for small apes. The femora in all small apes are straight in anterior view (this feature also is common in langurs). Moreover, the small-ape femoral shafts are straight in lateral view, as Dubois [65] recognized. Trinil 5703 has the straight anterior and lateral profile of small apes and langurs. We quantified the curvature in primate femurs and in Trinil 5703 through baseline registration (Figure 6). Trinil 5703 femur curvature is close to the genus *Presbytis* and *Hylobates* with a mean distance in baseline unit respectively of 0.010 and 0.014. The distance to the other primates is much greater, followed by *Trachypithecus* (0.037) and *Nomascus* (0.064), and finally the macaques *M. fascicularis* (0.073) and *M. nemestrina* (0.126). Moreover, there are features of Trinil 5703 that are seen in gibbons but not in other primates such as the shaft cross section morphology. While the cross section in female gibbons is circular, males have a spiral line, which resulting in an asymmetrical cross-section of the shaft. Trinil 5703 has a spiral line, although weakly developed.

Not all morphological details of Trinil 5703 fit those of modern small apes. In small apes of both sexes, the *linea aspera* is salient at the contact with the greater trochanter. This salience is not seen in Trinil 5703, neither at the contact of the greater trochanter nor more distally along the shaft. The salience of the *linea aspera* in Trinil 5703 is intermediate between those of small apes, macaques and langurs, falling within the 95% confidence intervals of the three groups. The weak development of the *linea aspera* in the femoral diaphysis of small apes is confirmed by shaft thickness (Figure 8). In keeping with these results, the index of platymeria for Trinil 5703 is 89.4 when measured below the lesser trochanter. A low platymeria index was found in gibbons by Manouvrier [66] (Table 2). Because Trinil 5703 is only partially preserved, a robustness index cannot be calculated for the specimen. Nevertheless, the shaft is thin and seems very elongated when considering its estimated length through both the macaque and small ape regression model.

Based upon the general morphology, shape and size of Trinil 5703, the specimen is a Hylobatidae. Nevertheless, some measurements on the fossil have values within the ranges of small apes, langurs and macaques. We employed log-shape ratios to clarify this issue. Trinil 5703 clusters with small apes using PC1 and PC2 in combination, which accounts for 64.57% of the total variance (Figure 9). A biplot of the PCA permits us to explain the clustering of the fossil by its very long and thin femoral neck (LNW and ANW variables), and by the discrete *linea aspera* on the shaft (ADW and ATW variables). In the LDA (Figure 10), the small apes are clearly separated from the three other natural clades — langurs, macaques and odd-nosed monkeys — by the first linear discriminant function (accounting for 88.82% of the total variance of the sample). The confusion matrix of the LDA (Table 4) shows a few misclassifications for the macaques clade, which plot with small apes. Indeed, only 14% (accounting for three individuals) of macaques are post-classified within small apes, while no langurs or odd-nosed monkeys have been misclassified as a small ape. These results increase the confidence that Trinil 5703 is a small ape.

## Discussion

The femoral neck-length and -height are the key parameters in establishing the taxonomic identity of Trinil 5703. These make an attribution to a primate the only reasonable one. These features, together with the general morphology of femur Trinil 5703 assessed by the metrical data, enable us to conclude that this specimen belongs to an adult or a subadult Hylobatidae.

Dubois' field crew collected Trinil 5703 while excavating the Trinil H.K. bonebed and catalogued the specimen as originating from the site. The color and state of preservation of Trinil 5703 are comparable to those exhibited by other fossils discovered in Trinil H.K., leaving little doubt that the specimen originated from the *Homo erectus* type stratum. Dubois [66, p.157] noticed that fossils from Trinil do not show prominent effects of fluvial transport. This is consistent with our own observations that Trinil 5703 is weakly abraded. From the geological evidence given above, as well as paleontological criteria [39], we conclude that the Trinil H.K. was deposited by a large river over a short period of time, with much of skeletal material in the bonebed apparently having come from deaths that had occurred shortly before deposition.

Although the femur Trinil 5703 had reached a practically full-grown stature, several features fit a subadult male: posteriorly positioned lesser trochanter, and weakly developed spiral line. Also the femur estimated original length is in the range of a present-day juvenile or sub-adult Hylobatidae, based upon metric comparisons of the fossil to modern material. Such a hypothesis could explain the presence of this isolated bone of a gibbon in Trinil assemblage, considering that high subadult mortality is not unusual for primates e.g. [68]. Alternatively, the Javanese Pleistocene is characterized by numerous changes in mammal body size, including dwarfism in large species and gigantism in small species (for an overview of the history, see [69]). Indications for this are for instance *Duboisia santeng* [54], *Mececyon trinilensis* [53] and *Stegodon trigonocephalus* [55]. Also, Hooijer [29] described a robust form of the small primate *Trachypithecus auratus* from the Trinil H.K. biozone. It is then possible that this gibbon evolved in isolation and therefore shows an unusual small size.

Gibbons are rare in the fossil record, and are found mostly as dentognathic remains [3]. The oldest examples of Hylobatidae in the fossil record are of Pliocene age from Yunnan Province, China. Only two fossils are known from the Lower Pleistocene also from China. No Hylobatidae fossil has been discribed from SEA before the Late Pleistocene Punung fauna of Java [3], but the primate specimens from the Punung fauna are teeth, only one of which is identified as *Hyolbates moloch* [4]. Ansyori [70] also identified an upper left second incisor of a *Symphalangus sp.* in the lower stratigraphical layers of Song Terus cave in the vicinity of Punung, and included the species in the Punung biozone. Trinil 5703, as a gibbon long-bone very likely attributable to the Lower/Middle Pleistocene Trinil H.K. fauna of Java, becomes the oldest known insular Hylobatidae fossil (on the insularity of the Trinil fauna, see [6]), one of the oldest representatives of the family in all SEA. Trinil 5703 further adds key skeletal material to the fossil record of a family that is principally known from dental specimens. The dating of Trinil 5703 supports the inference derived from molecular studies that gibbons first arrived in Sundaland near the Pliocene-Pleistocene boundary [71]. Lower/Middle Pleistocene fossils of Hylobatidae, which would be contemporaneous with Trinil 5703, have been recorded in China, Thailand and Vietnam [3].

Our identification of Trinil 5703 also provides key information on the paleoenvironment in the paleo-drainage area of Trinil. The Trinil H.K. represents remains of a once living community [39]. Although the predominance of herbivores in the assemblage indicates an open paleoenvironment, the lithological and paleontological features of the Trinil H.K. are better interpreted as representative of a broader set of paleoenvironments, including rain forest.

Paleoenvironmental studies have been conducted on the Javanese Lower/Middle Pleistocene deposits at Trinil and in the related lower Kabuh/Bapang sedimentary beds of Sangiran dome, 80 km to the west of Trinil. Study of the mammalian fauna, mainly from Trinil H.K., and the results of paleosol analyses at Sangiran indicate a savannah-like environment during this period [72]–[75]. Study of the ichthyofauna from Trinil H.K. gave similar results but also suggests the proximity of lakes and swamp forests. Based upon their study of the Trinil ichthyofauna, Joordens et al. [42] suggest that the grasslands were regularly inundated, and referred to the Trinil paleoenvironment as an “hydromorphic savanna”. Closed environment in the vicinity of Trinil during the Lower/Middle Pleistocene is also supported by the presence of the forest dweller bovid *Duboisia santeng* [54].

Palynological results from lower Kabuh/Bapang beds of Sangiran Dome testify to a mosaic of environments dominated by open vegetation (Poaceae) with ferns and rain forest-refuges [76, p. 131]. Palynological results from Trinil, although scarce, also testify to an open environment with the presence of a seasonal forest [76]. Fossil imprints of leaves and seeds of *Ficus* and *Indigofera* were collected from the Trinil discovery sequence by the Selenka expedition [77]. The fossils are considered to be typical of lowland rain forest [78].

The presence of a gibbon in Trinil H.K indicates that the closed forests were present near Trinil at the time of deposition. Small apes are mainly characterized by suspensory locomotion [79]. Their anatomy shows profound specialization for arboreal living, such as elongated limbs, including the hindlimb. In Trinil 5703, the globular femoral head, thin- and elongated-femoral neck, small-, deep- and rounded-trochanteric fossa, thin femoral shaft, and associated small platymeria index indicate that the species represented had suspensory locomotion comparable to that of present-day small apes. The diet of this clade largely consists of fruits and leaves. Acquiring these foods constrain small apes to high forest canopies, where dense tree branching is absent, and their preferred food resources are available throughout the year [80], [81]. Gibbons themselves are almost exclusively found in tropical lowland/upland evergreen rain forest. Mangrove, swamp and secondary forests are not appropriate for gibbons [80].

Evidence has been scarce in support of rain forest vegetation during Trinil H.K. [82], [83]. The fossil Trinil 5703 thus points to the presence of evergreen rain forest during the Lower/Middle Pleistocene of eastern Java. The rain forest in which the Trinil 5703 individual evidently lived was part of the drainage basin of the river that carried the femur to the site. The habitat might have been in distant volcanic highlands to the south, possibly higher than 1600 meters, which is the present-day upper altitudinal limit for gibbons in Java [84].

## Conclusions

The fossils that Eugène Dubois excavated from the Trinil site have drawn close scrutiny and often evoked controversy, because of their great importance to paleoanthropology. Some scholars have expressed concern about poor stratigraphic control during excavation, specifically that fossils from two geological formations might have been mixed [e.g., 85]. However, those who have looked closest at the provenience from archival and field evidence, including ourselves, conclude that the vast majority of the fossils Dubois collected at the site came from a single, one- to two-meter-thick bonebed, the Trinil H.K. [35], [5], [38], [36].

With our recognition of Trinil 5703 as the first Hylobatidae fossil from Trinil, we provide strong evidence that during the Lower/Middle Pleistocene the river basin upstream of the site contained a population of small-sized gibbons, who inhabited rain-forest, in addition to the *Homo erectus* and other mammals which have been known from the Trinil H.K. fauna for over a century.

## Supporting Information

Table S1 **Linear measurements used in this study.**
(XLS)

Table S2 **Shaft curvature 3D coordinates used for the baseline registration.**
(XLSX)
